# Pipeline Inspection Tests Using a Biomimetic Robot

**DOI:** 10.3390/biomimetics6010017

**Published:** 2021-03-09

**Authors:** Elizabeth Islas-García, Marco Ceccarelli, Ricardo Tapia-Herrera, Christopher R. Torres-SanMiguel

**Affiliations:** 1 Instituto Politécnico Nacional, Escuela Superior de Ingeniería Mecánica y Eléctrica, Unidad Zacatenco, Mexico City 07738, Mexico; eislasg1200@egresado.ipn.mx (E.I.-G.); ctorress@ipn.mx (C.R.T.-S.); 2 Laboratory of Robot Mechatronics, Department of Industrial Engineering, University of Rome Tor Vergata, 00133 Rome, Italy; 3 CONACYT Catedras, Mexico City 03940, Mexico; rtapiah@conacyt.mx

**Keywords:** biomimetics, service robots, spiders, pipelines inspection, design

## Abstract

This paper presents a biomimetic prototype of a mobile robot that can be used to inspect the subdrainage conditions of pipelines located along different highways in Mexico. Computer-aided design tools have been used to size each of the prototype components as inspired by anatomical spider structure. Springs are integrated to generate proper contact pressure against the pipe walls. The robot locomotion system is implemented with adaptable behaviour for the irregularities of pipelines along its journey. The robot prototype is manufactured in 3D printing with the advantage of having its spare parts easily replaceable. Reported results show internal pipe status through a mini video camera on the top of the robot.

## 1. Introduction

The locomotor behaviour and physiological characteristics of animals can reference robot development to perform different autonomously tasks under operator control. Robot design depends on the characteristics and conditions of the environment of its operation. Mobile robots’ movement is based on wheel type, caterpillar, legs, and worm [1]. Applications are on areas where humans are unable to access, for search and rescue in a natural event or disaster [2], inspection tasks [3], maintenance and cleaning [4], among others robotics service [5]. Most robots use wheels for their locomotion, achieving high speeds; however, they have disadvantages due to irregular travel areas [6]. The alternative of wheels locomotion is leg systems that are flexible and can avoid obstacles, also controlling the legs independently; they have multiple degrees of freedom [7]. Spiders inspire a climbing robot’s design with low energy cost and legs adhere to any surface walls [8]. 

As mentioned before, there are currently different robot applications, like a spider-shaped robot developed to support robot points used to move in flat tunnels. Its design is based on three limbs articulated in a central body, which determines posture stability and moves by stepping two legs on the tunnel walls while one moves [9]. Spider robot with eight legs was developed to move on flat surfaces, on vertical and inverted walls. Locomotor extremities parameters are stride frequency, joint twist angle, stride length, among others. Likewise, legs are symmetrical, which means that the legs’ behaviour is the same on both sides; speed movements depend on the frequency and stride length [10]. It is not always possible to imitate animals’ movements due to mechanical complexity high, and different morphology was implemented to design a spider robot with eight legs to avoid actuator consumption. This actuator and the single-degree-of-freedom belt-pulley mechanism design make all eight legs move forward through the transmission integration. This robot’s primary purpose [11] is to traverse smooth terrain and check a tetrapod gait pattern. According to different investigations as in [12,13], robots have also been developed with hybrid locomotion; a propulsion mechanism combination; caterpillar wall press type, wheeled wall press, and wheel wall pressure screw-type. A design for gas supply pipes is presented in [13], with a mechanical structure that allows moving with elbows, by a sufficient traction force and flexibility in their travel. It has three-dimensionally differential drive wheels, which can be adjusted inside the pipe, making the wheels drive contact against the wall. The robot was designed with two V-shape mechanisms, capable of sliding in smooth polyvinyl chloride (PVC) pipes between 63 to 125 mm diameters. It performs an axial rotation inside the tube with clamping movements [14]. Friction force generated using this V-shape depends on the tube diameter. Other results [15] show the design and construction to inspect pipeline systems in residential buildings with length of more than 30 mm. The design efficiency was verified through a computational modelling tool. In addition, [16] exhibits a robot to cut pipes in decommissioning of nuclear plants. The structure was made under radioactive conditions, it is composed of vision, temperature, and position sensors, and a mechanism was integrated for the cutting task. A robot’s design to inspect pipes vertically, horizontally, and in elbows that are related to petrochemistry, water supply, and liquid transport with dimensions from 127 to 152 mm in diameter is shown in [17]. Its mechanism includes a module with three wheels in a vertical position, with the position and three wheels attached at a propeller angle of 15° perpendicular to the body, the rotation of this coupling generates the robot movement. The robot in [18] is developed to inspect pipes in the chemical and gas industries. It is composed of a system of front and rear legs coupled to a springs system, positioned at an angle of 120° to each other. Through the springs attached to each leg and to the robot body, it is able to move inside pipes of a range from 140 to 200 mm. Some developed robots can adapt to different changes in pipe diameters and apply gas systems [19,20,21]. At the same time, other results [22,23,24] were designed for a specific diameter of pipes. Results in [25] show a robot with dimensions of 23 mm in diameter and 110 mm in length was developed. Inspecting petrochemical pipes with the integration of a small camera, recover lost objects with micro arms. Pipes are an essential component to transport different fluids or gases from one point to another, requiring continuous maintenance [26]. Everyday monitoring of highways in Mexico is aimed to avoid and correct pipe damage [27]. Subdrainage networks are a significant part of releasing rainwater and preventing pavement fractures and, most importantly, preventing car accidents.

This paper presents a robot prototype to inspect subdrainage pipes networks that are located along different highways in Mexico. It is essential to find the exact point where a pipeline present failures and obstructions, causing water stagnation under the roads to prevent pavement fractures. Obstructions can be caused by the dragging of small objects that exist around highways. The robot’s design integrates omnidirectional locomotion to avoid some obstacles and unrestricted movement (rotation and translation) through the pipe, in addition to manufacturing by 3D printing of polylactic acid (PLA) filament which reduces the total cost compared to a commercial robot. The advantage is the easy replacement of components. This paper is organized as follows: The Methodology section shows a proposal for locomotion and the type of wheels. The Materials are also considered to obtain adequate traction; the Result section shows a robot design capable of moving and rotating through a PVC pipe. The conclusions summarize the requirements for highway inspection in Mexico.

## 2. Methodology

The robot is introduced into subdrainage networks with a diameter between 3 and 4 inches of approximately 100 m of length. Due to these characteristics, it is not possible to visualize the interior by conventional means such as the visual reach of the human being or commercial robots. The inspection aims to visualize the inside of the pipe using the night vision camera. To take photographs with a minimum resolution of 5 MP, to know the conditions they were found, and determine if immediate maintenance was required. The robot will inspect the pipeline in sections of 50 m. Table 1 shows robot data whose design is inspired by a spider structure and locomotion by wheels as references for this work. Likewise, it highlights that this proposal has a free movement within the pipe than the mentioned references.

**Table 1:** Hexapods designed for pipe inspection.

Robot	Use	Characteristics	Design Requirements	Pipe Size
Three-limb spider-like robot [9]	Inspection of planar tunnel environments.	Algorithm for selecting the footholds of a three-limb robot. The algorithm assumes knowledge of the tunnel geometry.	No mention of component replacement. It does not avoid obstruction.	90–100 cm
The ROBOTURK SA-2 Robot [11]	Applications in industry, defence, and natural disasters.	Design and control of an eight-legged with a single actuator.	Avoid obstacles but not in pipe. No mention of component replacement	-
The MRINSPECT IV robot [13]	Gas pipe inspection.	Design to straight pipelines, elbows, and branches and different diameter pipes.	Only translational movement It does not avoid obstruction. No mention of component replacement.	8.5–10.9 cm
The Pirate robot [14]	Inspection of low-pressure gas distribution networks.	Using omnidirectional wheels that allow direct control of the orientation in the pipe.	Control automatic of movement translational and rotational It does not avoid obstruction.	6.3–12.5 cm
New in-pipe inspection robot [17]	Inspection petrochemistry, water supply, and liquid transport pipes	It uses wheels in a vertical and horizontal position. Horizontally or vertically pipe, straight pipelines and elbows.	It does not avoid obstruction. Movement and rotational only one module	12.7–15.2 cm
Inspection Robot CAD [18]	Inspection gas pipelines, water pipelines and drain pipes	Pipe horizontally or vertically, straight pipelines and elbows. 2 motors for 8-wheel movement. Use springs to adapt to different diameter.	Only for translational movements It does not avoid obstruction.	14–20 cm
Robot “MORITZ” [24]	Inspection gas pipelines	Climb through pipes of different inclinations. Can manage tube junctions.	Not mention if avoid obstacles. Only for translational movements.	60–70 cm
Micro Inspection Robot [25]	Inspection for 1-in Pipes in the chemical industry	A dual hand for manipulating small objects in pipes. Developed several microdevices and micromechanisms.	It does not have movement rotational. It does not avoid obstacles.	2.54 cm
Abigaille II spider-like robot [28]	Flat vertical surfaces inspection.	Able to climb vertical surfaces and surfaces of any inclination.	Avoid obstacles but not in the pipe. Only movement translational.	-
Robot with camera compensation [29]	Inspection water supply pipeline	Passive adaptation ability. A different algorithm is tested to compensate the camera image rotation.	Control automatic of movement rotational. No mention of component replacement.	12–18 cm
Robot hybrid, leg and wheels [30]	Inspection plastic or metallic pipes	Operate inside pipes of different diameters.	It does not avoid obstacles. Only movement translational.	12.5–18 cm
Kinematic modelling robot [31]	Inspection gas pipelines	Having three caterpillar wheel chains. Pipeline with elbows.	It does not avoid obstruction.	10–12 cm

According to the articles mentioned above, they do not meet the requirements to inspect pipelines. The proposal with omnidirectional wheels will allow free movement (rotation and translation) within the pipe, managing to avoid obstacles. A low-cost will be obtained in the prototype’s manufacture due to the 3D printing and the parts’ easy replacement. The structure is the star type. It has modules every 120° while the proposal will place modules every 180°.

**Figure 1:**
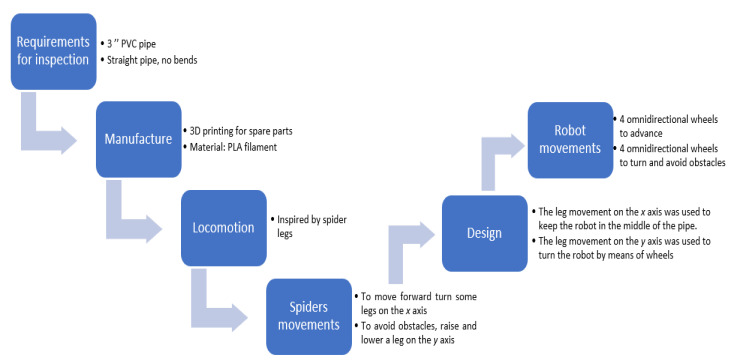
Design concepts for mobile robots as inspired by spider structure.

Mobile robots with legs have many degrees of freedom than other locomotion, but they consume more energy because the number of movements is more significant to generate displacements. In addition, maintaining stability is complicated due to the lack of contact; however, the advantage is that they can move over almost any terrain. The mechanisms with chains present inaccuracy in the odometry since they can slide on the land accumulating position error on a starting point. The wheeled mechanisms construction is relatively simple and presents stability and direction changes compared to other locomotion systems [32]. Figure 1 illustrates a block diagram to explain the designs as inspired by spider structure. First, it was considered in the requirements for the inspection defining the pipe characteristics; in the manufacturing, 3D printing was contemplated to have spare parts if a piece suffered a fracture, in addition, the spider’s movements were observed to define the type of locomotion and design. Finally, the movements of the robot were defined using the omnidirectional wheels.

**Figure 2:**
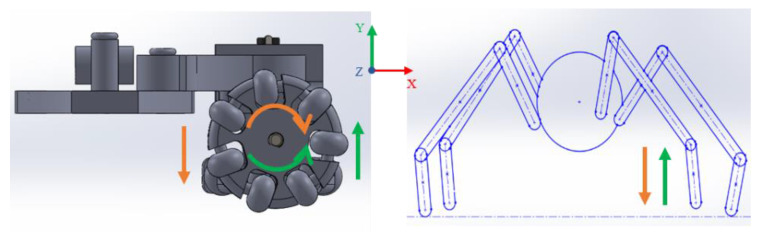
Robot movements are imitating a spider in the *y*-axis direction.

Pipes space reduces the possibility of free robot movements. Therefore, the robot’s design is based on hybrid locomotion, where they have legs and wheels; the legs influence the robot to stay in the middle of the pipe while the wheels help the movement. Subdrainage pipes accumulate dust in their lower part, so four lateral legs with springs attached to the body can be conveniently implemented. Furthermore, the legs can be actuated with a pressure that is exerted by springs. Figure 2 shows a proposed design referring to spider characteristics in which the attached body links are lifted in the *y*-axis, while the entire body robot rotates on its axis. Each arrow represents the direction in which the spider’s legs and the wheels of the robot move. A robot needs these movements to avoid obstacles.

**Figure 3:**
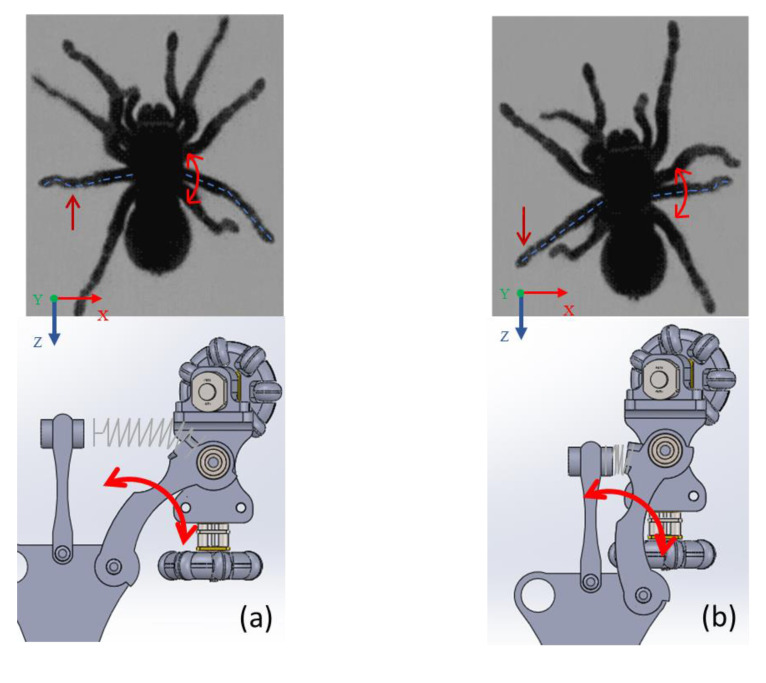
Movements of a spider and a robot design along *x*-axis direction (**a**) The robot leg imitating the spider leg’s movement when it is out of the pipe (**b**) the robot leg imitating the movement of the spider leg when it is inside the pipe.

Figure 3 shows that the robot’s legs imitate the spider’s leg; this influences the movement; it keeps it halfway down the pipe. The same movement is generated when the robot is outside, and when it is introduced, it adjusts to the pipe’s diameter. Furthermore, it shows the links attached to the robot body with rotation capability around the *x*-axis with a spring-actuated link. This spring links exerting pressure against the pipe’s walls, keeping the robot halfway across the pipe while moving forward. Wheel movement is specified in Figure 3 as executing a *z*-axis forward robot motion with a comparison in which a spider performs similar movements for a similar function. However, not all horizontally positioned wheels have the same rotation for the robot to move forward or backward, so it is designated that the wheels on the right side rotate clockwise while the wheels on the left side rotate clockwise on the opposite side.

In order to know the spring force with which pressure is exerted on the walls of the pipe, the following procedure was carried out. Dimensions of the length of the tension spring were taken by placing different weights, to calculate the spring’s elasticity constant, an average of these approximate constants was obtained. Later, the force was calculated with the mathematical expression *F = −K ×* ∆*x*.

**Figure 4:**
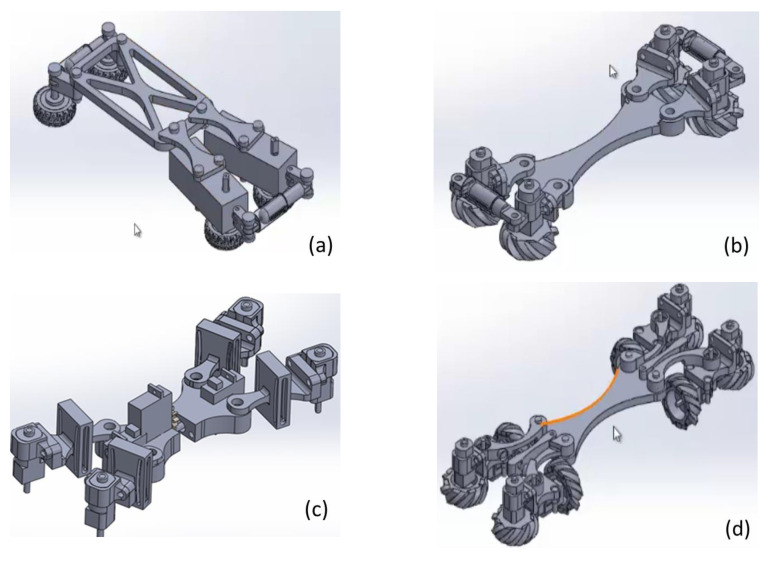
Mechanical design sketches; (**a**) Power transmission with two gear motors. (**b**) Power transmission with four micro gear motors and springs. (**c**) Mechanism with a servomotor to control the position of the robot. (**d**) Design with eight wheels in a horizontal and vertical position.

Different mechanical designs have been attended for a pipeline robot for inspection application. Figure 4a shows the first sketch with gear-motors placed on two legs and springs added in an original design at the front and rear to press the wheeled feet against the pipe walls. However, there was not enough force for the displacement. However, in this work, precision in robot movements is considered more important than speed and therefore, four micro-gear motors have been used to reduce the speed and increase the actuating torque. Thus, the size of the robot legs has been reduced, as shown in Figure 4c. During preliminary tests, a system was placed in the middle of the robot’s base to rotate the mechanism with a servomotor. Likewise, other servomotors were used to replace the springs in the pressure system to control the moment in which pressure is exerted or not and rotate the mechanism to help position the robot in the pipe at will. However, the servo motor was not strong enough to rotate the robot’s body. Therefore, it was proposed the horizontal and vertical wheel in each leg. A total of eight wheels and eight microgear motors are used to improve traction, as shown in the solution in Figure 4d as a final design.

**Figure 5:**
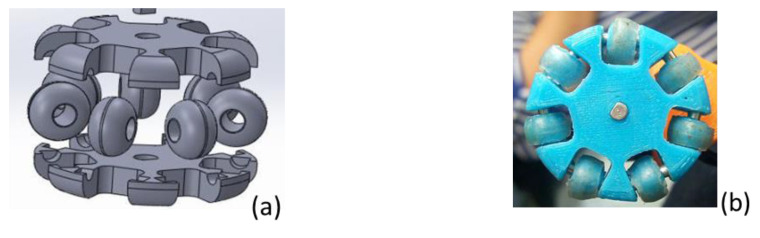
Universal omnidirectional wheel for pipeline robot; (**a**) Cad Model. (**b**) Omnidirectional wheel with thermofit.

A wheeled feet design began with conventional embossed wheels contacting the wall pipe, for which there was a lack of traction, causing a skid on the smooth surface of a PVC pipe. The material of these wheels was PLA. A final design is focused on using universal omnidirectional wheels, achieving the robot’s rotation inside the pipe with control displacement conditions. It is essential to mention that commercial wheels are not used because the small size is required for application in 4-inch diameter pipes. In Figure 5a, the final wheel design is shown with the roller’s design perpendicular to the wheel’s periphery by eliminating the friction that may exist because of movements and allows displacements in both directions. Heat shrink tubing on the wheels’ rollers was used as in Figure 5b, to reduce the roller diameter to slightly less than half of its initial diameter before starting to burn or melt. Plastic material complies with plasticity and flexibility characteristics for traction, and its contracts give the advantage to adapt the wheel diameter without affecting its structure.

The following procedure is worked out to obtain the load that the robot supports, using a force diagram in which the motor’s weight, the friction forces, normal and the force of the robot’s movement were considered as in the following.

**Figure 6:**
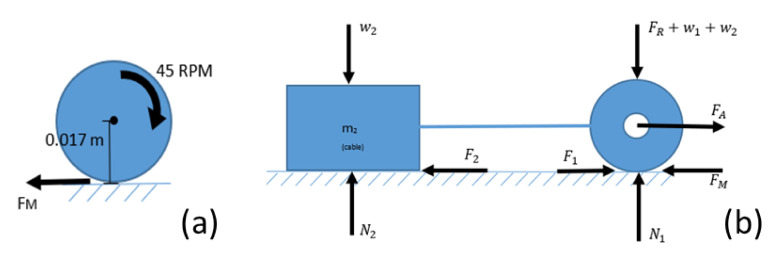
(**a**) Diagram to obtain the tangential force. (**b**) Force diagram to obtain the load the robots support.

The robot has 4 points at which equal forces are applied to make it move. The tangential force for the robot’s movement is obtained, as shown in Figure 6a. It is necessary to know the pulling force that the robot generates since, in addition to itself, it must carry enough cable to run through the pipe sections. 

According to the force diagram Figure 6b, regarding the wheels, the forces in ‘Y’ and ‘X’ are obtained:

$$\sum^{\;}Fy=0\;-F_{R}-w_{1}+N_{1}=0\;N_{1}=F_{R}+w_{1}\;\;=4.6\;N\;\sum^{\;}Fx=ma_{x}\;-F_{M}+F_{1}+F_{A}=m_{1}a\;F_{A}=m_{1}a+F_{M}-F_{1}=14.88N.$$

For Figure 6b, which represents the weight of the cable, we have:

$$F_{2}=µN_{2}=\left(0.402\right)\left(9.81\frac{m}{s^{2}}\right)m_{2}=3.9436m_{2}\;\sum^{\;}Fy=0\;-W_{2}+N_{2}=0\;N_{2}=W_{2}\;\sum^{\;}Fx=m_{2}a_{x}\;F_{A}-F_{2}=m_{2}a\;F_{A}=m_{2}a+F_{2}.$$

We substitute, $a=0.0811\;m/s^{2}$

$$14.8837\;N=m_{2}\left(0.08011\;m/s^{2}\right)+3.9436m_{2}.$$

Considering the analysis was made in one of the four support points of the robot: Theoretically, it is the total weight that the robot can drag: $m_{2}\times \;4\;=\;14.79\;kg$. The maximum length of cable is 50 m with an approximate weight of 5 kg.

### 2.1. Robot Movement Control

**Figure 7:**
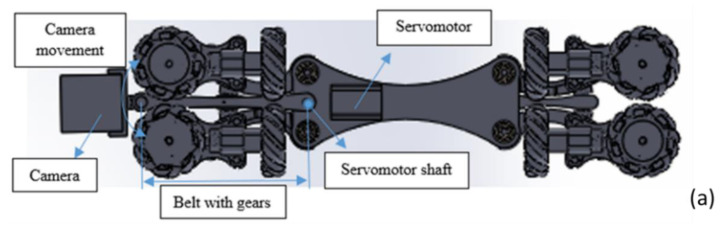

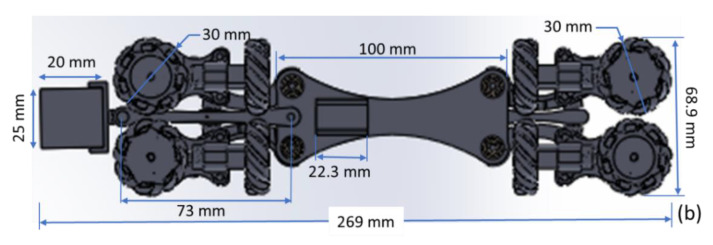
Components of the proposed pipe robot (**a**) Servomotors and camera position. (**b**) Robot dimensions.

In the final prototype (Figure 7a), it is observed how each piece is assembled with others, since the motor is placed with a vertical axis to move forward and backwards easily, and in the horizontal axis, so the robot could also rotate around its axis. A camera with night vision capability was installed to visualize the pipe’s interior in real-time and take pictures where there will be an obstruction, out of phase or fissures. It uses a mini night vision camera for remote and real-time monitoring with Wi-Fi wireless communication technology, with dimensions of 2.2 × 2.5 × 2 cm (height × width × depth). In real-time, the video will be transmitted through a screen outside the pipeline, where the data necessary to decide on maintenance will be archived. The protocol (IP) is used for data transmission through an Acer Aspire 5 laptop, and if the transmission is lost, the camera has the ability to continue recording and save the video in a microSD memory. Figure 7a shows servomotor coupling at the base of the robot and a belt with gears to transmit the camera’s movement, and Figure 7b shows the size of the main components. The camera is located on the front of the robot. Without attaching the springs, the dimensions of the robot are 68.8 mm wide and 269 mm long. The diameter of the omnidirectional wheels is approximately 30 mm.

**Figure 8:**
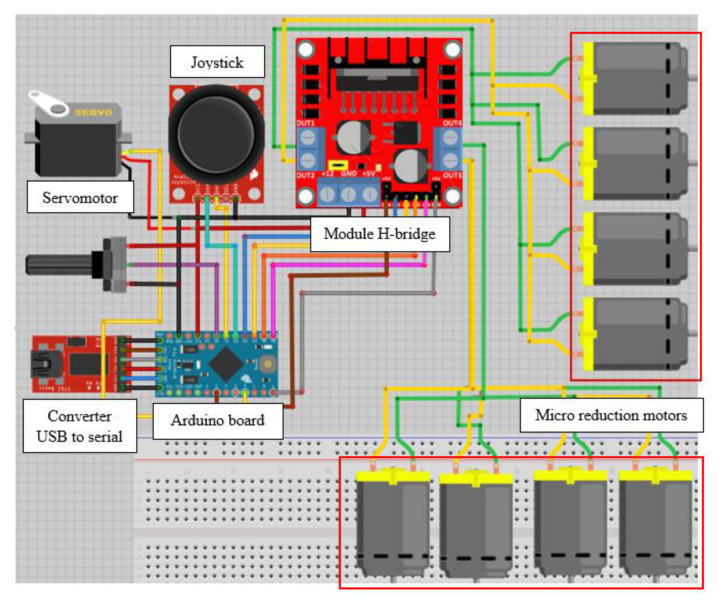
Control hardware design for the proposed robot in Figure 6.

Robot controlled movements are generated from software installed with all electronic components (forward, backwards or rotate, and left or right). Figure 8 shows the converter USB to serial linked to the Arduino^®^ card to download the code to this card. Analogy inputs of the Arduino^®^ card are connected to the joystick, while this card’s outputs are connected to motors and servomotor. Through an Arduino^®^ card, the robot movements are produced by a joystick device composed of two analogue potentiometers. This card regulates the pulse width modulation (PWM) of the motors that control their speed in an open loop. The control code is designed to execute the movements of the design pipe robot. When potentiometers are at halfway, the robot must remain in stationary configuration, and in other motion ranges, the joystick moves in one direction so that the robot moves or rotates. Motor speed control depends on the path of the potentiometer, and displacements imply the change of direction of rotation of the motor, so the card with an arrangement of transistors and diodes is correctly used, allowing the control of the polarity of the output terminals. The potentiometer’s function in Figure 8 is to control the camera’s movement by the servo motor. The camera remains centred when the potentiometer is half the value (ohms). Therefore, when it is in other ranges of the potentiometer, the camera will rotate to the left or right. Moreover, in Table 2 shows its characteristics of components for movement control of the proposed robot.

### 2.2. Numerical Analysis

**Figure 9:**
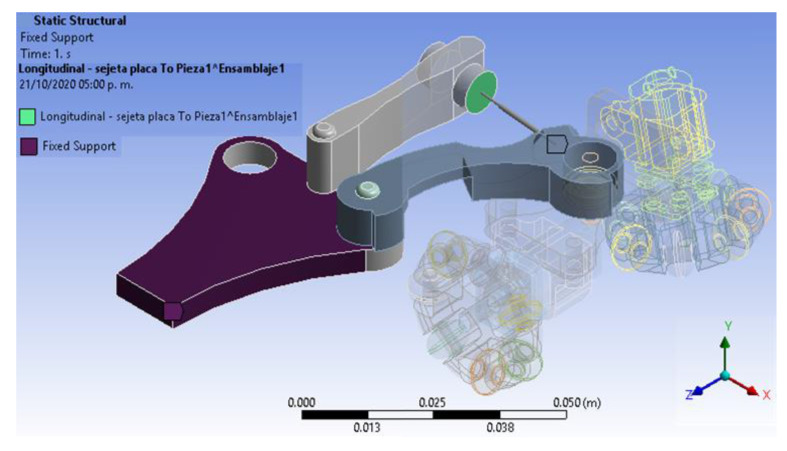
Boundary conditions, fixed supports.

Static analysis has been worked out to determine and characterize the structural behaviour due to external loads in the leg pressure against the pipe wall exerted by the spring when entering the pipe. This analysis only applied for a quarter part of the designed robot because its limbs have the same setup and dimensions. Additionally, the boundary conditions have been defined, as are shown in Figure 9. Moreover, the fixed support point on the robot body is observed because it only supports the other components, and it does not have any movement. A connection is made with spring conditions (longitudinal) on the green painted faces as shown. Likewise, the elasticity constant of this element was determined, along with the connection with spring conditions. The complete geometrical design meshed into 38,376 elements with 109,877 nodes for finite element analysis (FEA).

### 2.3. Pipeline Inspection Tests

**Figure 10:**
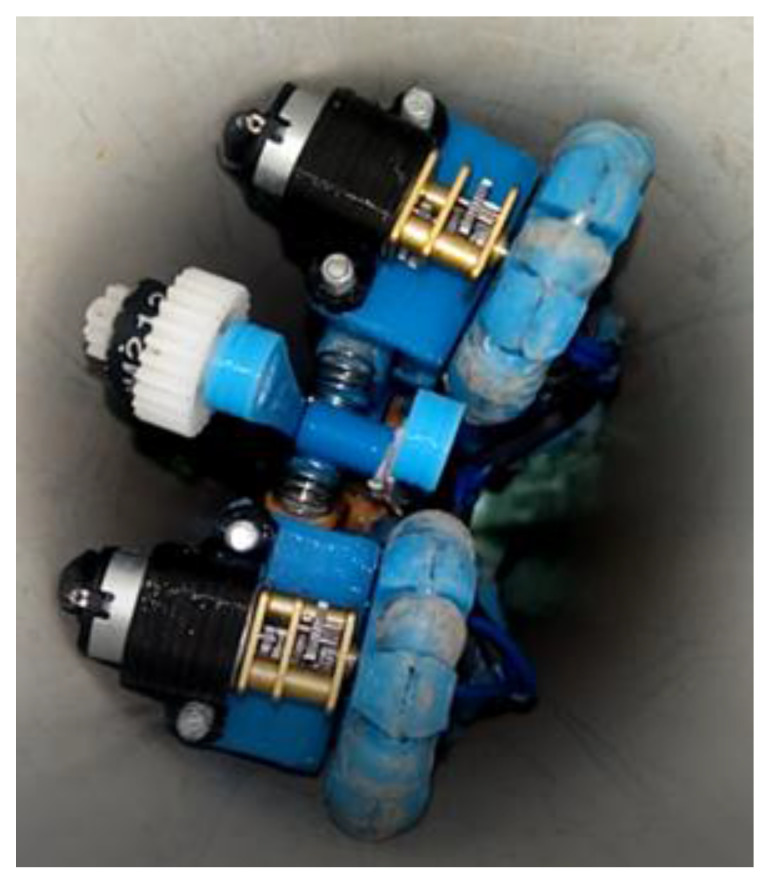
Control robot position due to the joystick and the omnidirectional wheels.

Each of the proposed designs’ behaviour is observed, the robot was subjected to an experimental test inside a 2 m long PVC pipe. As previously mentioned, two geared motors simulating two legs and two springs were added to the robot’s body at the front and rear, applying pressure to the pipe walls. The traction was considered more important in this work, so we changed to micro geared motors inside this system. It provoked a rotation inside the pipe. Consequently, an omnidirectional wheel was placed in each position, horizontal and vertical. Figure 10 shows the robot’s position; therefore, it is possible to avoid the pipe’s obstacles thanks to the omnidirectional wheels’ control.

## 3. Results

**Figure 11:**
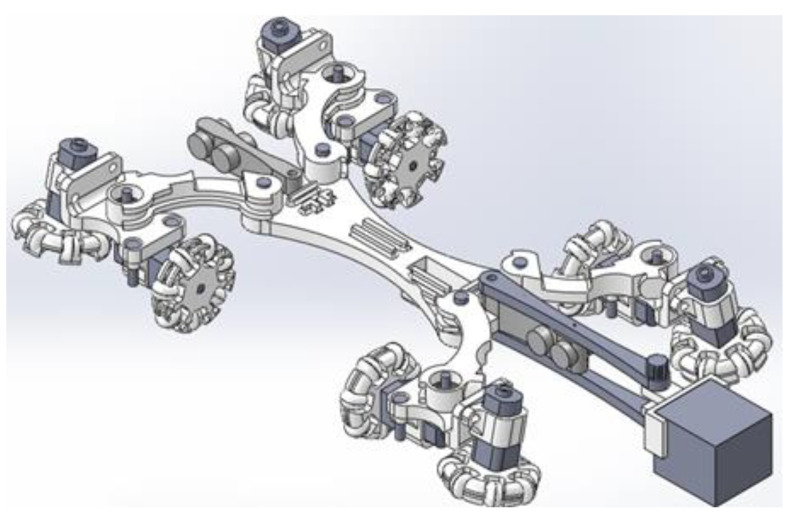
3D representation of the proposed pipe robot.

Figure 11 shows the final robot design in 3D representation used in pipeline inspections and performs the structural analysis through the finite element method. All the parts of the prototype have been assembled with the help of screws and bearings in a prototype, connecting motors that are located with their respective omnidirectional wheels and the camera structure.

**Figure 12:**
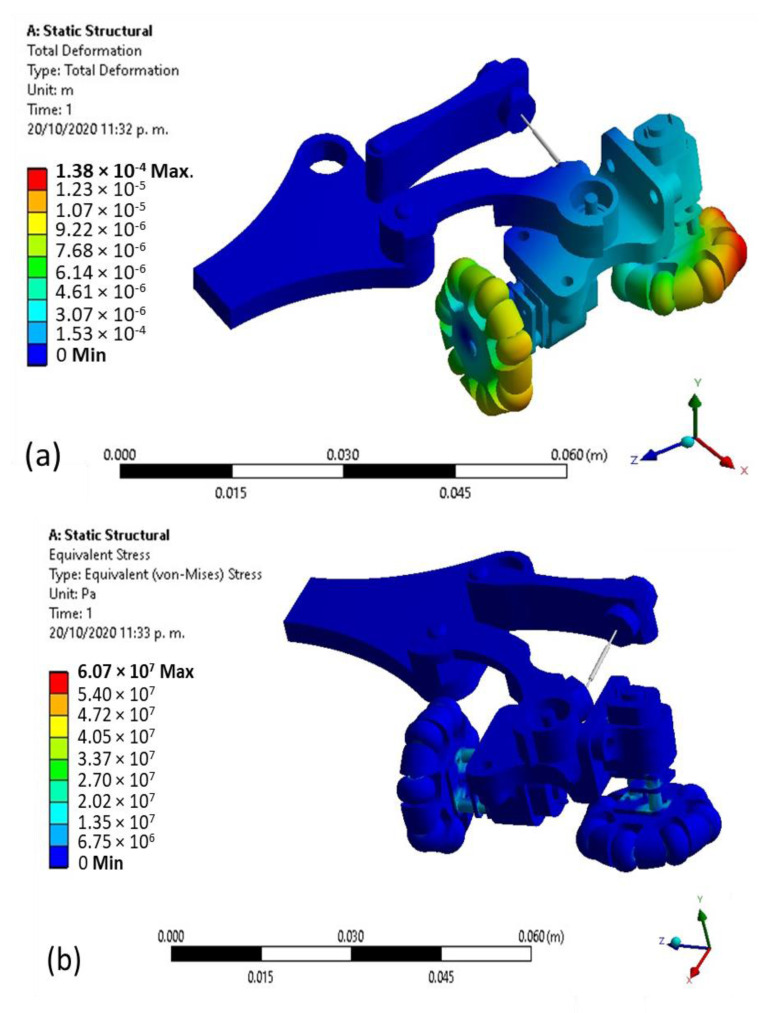
Computed results from structural analysis (**a**) Total deformation. (**b**) Equivalent stress.

The results that are computed by a structural analysis are summarised in Figure 12. Figure 12a deformation of the robot’s 3D model concerning its original geometry is shown from computations using a strength applied in the wheels and von Mises failure criterion as per the used ductile material. The maximum deformation can be observed in the omnidirectional wheel’s rollers, while the minimum deformation is in the part that holds the motors. The wheels are the only piece that has contact with the pipe walls and therefore receives the spring’s pressure. Moreover, the maximum deformation is calculated as 1.38 × 10^−5^ m in the wheel’s front. The maximum stress occurs in the motors area near the axis as shown in Figure 12b where the maximum stress refers to an element with 6.07 × 10^7^ Pa.

Electronics for control movements are located outside of the pipe, so engines suffer a cable load and robot weight. Enough cable length is needed to run through the pipe. The normal force that supports the cable and the robot’s mass, the friction force and the robot motion’s net force are involved. The total robot weight is capable of dragging 14.79 kg.

By the characteristics of the pipes and requirements mentioned above, a small portable video camera with infrared night vision light can be installed on the robot body to visualize the pipeline in real-time. A screen for monitoring control is installed outside the people under inspection. Camera movement from left to right has been programmed within the control code for actuating a servomotor. The solutions mentioned above are tested as robust according to the requirements; however, there are still disadvantages such as the microgear motors’ dimensions, preventing additional robot size reduction. Working with pipes for stormwater discharge requires airtightness for the actuators, although the coverage with thermofit is insufficient to attack this problem.

**Figure 13:**
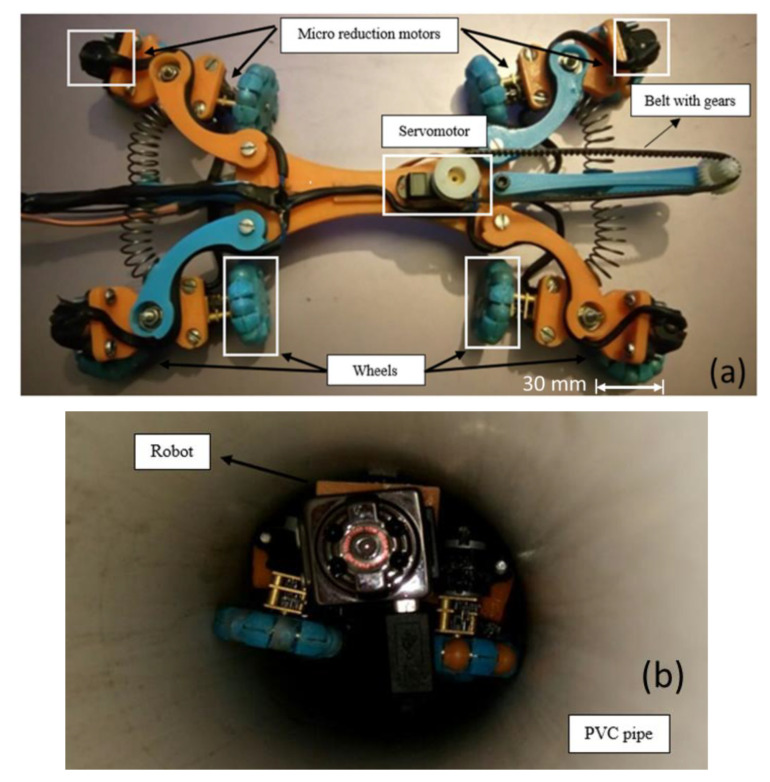
Robot prototype (**a**) 3D printing parts. (**b**) Robot inspection inside polyvinyl chloride (PVC) pipe by camera.

3D printing parts for constructing the robot prototype and all electronic components are shown in Figure 13. The wheel dimension is also shown for a closer look at the robot dimensions. Moreover, it shows the robot inside the PVC pipe. After printing each part of the robot, they were joined by means of screws and bearings, depending on whether it was a joint or if the part should be fixed. The servomotor is placed in that position so that the belt with gears will adjust with the gear and transmit the movements of the camera. The springs were also placed on the legs of the robot and the omnidirectional wheels to each motor.

Moreover, the motors’ connections were made with a cable fixed to the parts, as shown in Figure 13a. The robot underwent physical tests inside the 3 inch diameter PVC pipe in its different construction stages, as shown in Figure 12b. Points were established where improvements should be made to satisfy the inspection. It is also observed that the robot occupies most of the space inside the pipe. Reducing the size requires a smaller camera and motors.

## 4. Conclusions

The design of a mobile robot with omnidirectional wheels is proposed to be used only in visual inspection tasks on straight 7.6–10.16 cm PVC pipes without curves. It includes tasks for moving within pipes in which the subdrainage accumulates dirt in their lower parts. With the proposed solution, if there is an obstruction, the robot cannot clean the pipe, it only avoids the obstructions. The attached night vision camera met the requirements, taking real-time video and 5 MP photographs to present the pipe’s conditions.

Therefore, it was possible to design an appropriate robot for the indicated requirements, adapt to pipes of different diameter, avoid obstacles by turning the robot around its axis within the pipe, low-cost manufacturing around $600 dollars, obtain images through the screen for later analysed the conditions of the sub-drainage networks of the different highways in Mexico.

According to the theoretical development, the robot should be able to drag 50 m of cable; however, it is intended to carry out, in the future, experimental tests to verify the theoretical results. 

Experimental results have verified that wheel traction for its movement is limited by the robot’s tendency to rotate around its axial axis. An omnidirectional wheel system is implemented in each leg, allowing the corresponding displacement correcting the robot’s orientation. Likewise, the electronics for the control is designed for the movement of the robot inside the pipe. The heat shrink tubing in the wheels’ rollers achieves adequate traction, so the robot does not skid when advancing. However, when the material pipe suffers wear, the friction coefficient may decrease radically, causing the wheels skidding on the PVC by affecting the robot’s speed and drag force due to the pipes’ dirt and dust conditions.

The structural analysis through finite element analysis (FEA) shows deformations and maximum stress in a 3D model of the robot prototype that can be sued to prevent failures when the robot prototypes are used to inspect pipes. Finally, it is necessary to reduce mechanical elements that integrate the robot to increase the robot wheels’ useful life and implement an odometer that allows knowing the robot’s location inside the pipe.

## Figures and Tables

**Table 2:** Hardware and software components.

Components	Characteristics
Arduino^®^ card	ATmega328 microcontroller operating at 8 MHz 14 digital inputs/outputs 6 analogue inputs Maximum output current: 150 mA Dimensions 18 × 33 mm
Joystick	Two independent 10 K potentiometers: one for each axis (*X* and *Y*) Button (pushbutton) on the *Z*-axis Operating Voltage: 3.3–5 V DC Automatic return to the center position Compatible to interact with Arduino^®^ card
Module H-bridge	It has 2 independent H-bridges Main Integrated Circuit: L298N Peak operating current: 4 Amps Constant operating current: 2 Amps Motor supply voltage up to 24 volts
Converter USB to serial	Compatible with Arduino cards Get access to GND, CTS, VCC, TX, RX, and DT signals
Micro Metal Gearmotor LP 6V	Speed: 45 RPM. Supply voltage: 6 V Current without charge: 40 mA Maximum current: 360 mA Maximum torque: 2.9 kg/cm
Servomotor	Dimensions (L × W × H) = 22.0 × 11.5 × 27 mm Torque at 4.8 volts: 16.7 oz/in or 1.2 kg/cm Operating voltage: 4.0 to 7.2 volts Speed at 4.8 volts: 0.12 s/60°

## Data Availability

Not applicable.
